# Endoscopic Diagnostics for IgG4-Related Pancreatobiliary Diseases: Current Modalities and Clinical Perspectives

**DOI:** 10.3390/diagnostics15161990

**Published:** 2025-08-08

**Authors:** Itaru Naitoh, Michihiro Yoshida, Takahiro Nakazawa

**Affiliations:** 1 Department of Gastroenterology, Nagoya City University Midori Municipal Hospital, Nagoya 458-0037, Japan; 2 Department of Gastroenterology, Nagoya City University Graduate School of Medical Sciences, Nagoya 458-0037, Japan; mityoshi@med.nagoya-cu.ac.jp (M.Y.); nakazawax3@gmail.com (T.N.)

**Keywords:** autoimmune pancreatitis, endoscopic retrograde cholangiopancreatography, endoscopic ultrasonography, IgG4-related cholecystitis, IgG4-related disease, IgG4-related pancreatobiliary disease, IgG4-related sclerosing cholangitis, intraductal ultrasonography

## Abstract

Type 1 autoimmune pancreatitis (AIP), IgG4-related sclerosing cholangitis (IgG4-SC), and IgG4-related cholecystitis are recognized as IgG4-related pancreatobiliary diseases. Endoscopic retrograde cholangiopancreatography (ERCP) and endoscopic ultrasonography (EUS) are crucial diagnostic modalities for these conditions. In the diagnosis of AIP, EUS-guided tissue acquisition plays an important role in obtaining histological confirmation and excluding pancreatic cancer (PC). EUS, including contrast-enhanced harmonic imaging and elastography, is used to differentiate focal-type AIP from PC. Endoscopic retrograde pancreatography (ERP) is utilized to obtain a pancreatogram when it is challenging to distinguish AIP from pancreatic cancer. Duodenal papilla biopsy may serve as a supplementary tool, particularly in cases involving the pancreatic head. Cholangiographic classification is essential for differentiating IgG4-SC from PC, primary sclerosing cholangitis (PSC), and cholangiocarcinoma (CCA). ERCP is commonly performed for additional ERCP-related procedures. Intraductal ultrasonography (IDUS) is useful for distinguishing IgG4-SC from CCA or PSC. The primary role of bile duct biopsy is exclusion of malignant biliary strictures; EUS-guided tissue acquisition may also provide histological evidence of IgG4-SC. In the diagnosis of IgG4-related cholecystitis, EUS is helpful to differentiate it from gallbladder cancer. EUS-guided tissue acquisition can aid in confirming IgG4-related cholecystitis and excluding gallbladder cancer or xanthogranulomatous cholecystitis. Transpapillary gallbladder cytology or biopsy may also be considered. Overall, endoscopic modalities play a critical role in diagnosing IgG4-related pancreatobiliary diseases.

## 1. Introduction

IgG4-related disease (IgG4-RD) is a systemic condition characterized by infiltration of abundant IgG4-positive plasma cells with fibrosis, typically accompanied by elevated serum IgG4 levels [1,2]. Multiple organs can be affected by IgG4-RD. Type 1 autoimmune pancreatitis (AIP), IgG4-related sclerosing cholangitis (IgG4-SC), and IgG4-related cholecystitis are classified as IgG4-related pancreatobiliary diseases. IgG4-RD generally responds well to steroid therapy. Japanese clinical diagnostic criteria and guidelines have been established for AIP and IgG4-SC; they are utilized in the diagnosis and management of these diseases [3,4,5,6,7]. In contrast, the pathogenesis and clinical features of IgG4-related cholecystitis have not been fully elucidated. To date, several case reports and small case series have described localized forms of this condition.

Endoscopic modalities play a central role in diagnosing pancreatobiliary diseases. Endoscopic ultrasonography (EUS) and endoscopic retrograde cholangiopancreatography (ERCP) are two major modalities used in the diagnosis of IgG4-related pancreatobiliary diseases [8,9,10,11,12,13]. The European guideline on IgG4-related digestive diseases emphasizes the importance of EUS in the diagnosis of AIP and IgG4-SC [14]. EUS-related procedures include EUS-guided tissue acquisition (EUS-TA) methods, such as EUS-guided fine needle aspiration (EUS-FNA) and EUS-guided fine needle biopsy (EUS-FNB). ERCP-related procedures incorporate biliary intraductal ultrasonography (IDUS), transpapillary bile duct or gallbladder cytology and biopsy, and duodenal papilla biopsy. This review aims to delineate the current roles of various endoscopic modalities in the diagnosis of IgG4-related pancreatobiliary diseases.

## 2. Methodology

A comprehensive literature search was systematically conducted using the PubMed, Embase, and Cochrane Library databases. In addition, the reference lists of the retrieved articles were manually screened to identify further relevant studies. The search was restricted to English-language publications involving human participants and published up to June 2025. The following search terms were used: “autoimmune pancreatitis” or “IgG4-related sclerosing cholangitis” or “IgG4-related cholangitis” or “IgG4-associated cholangitis” or “IgG4-related cholecystitis”.

## 3. Autoimmune Pancreatitis

### 3.1. Clinical Overview

AIP is classified into types 1 and 2 in the International Consensus Diagnostic Criteria for AIP (ICDC) [15]. Type 1, characterized by the histological features of lymphoplasmacytic sclerosing pancreatitis, represents pancreatic involvement of IgG4-RD [16,17,18]. In contrast, type 2, defined by idiopathic duct-centric pancreatitis and granulocytic epithelial lesions, is pathologically distinct from type 1 AIP [19,20,21]. In this review, AIP refers to type 1 AIP unless specified otherwise.

AIP typically presents with pancreatic enlargement and irregular narrowing of the main pancreatic duct (MPD) on imaging. Pancreatic cancer (PC) is a key differential diagnosis. In Japan, AIP is diagnosed using the Japanese Clinical Diagnostic Criteria for AIP 2018 (JPS2018) [5], the ICDC [15], and the Japanese Consensus Guidelines for AIP 2020 [7]. The JPS2018 includes six criteria: enlargement of the pancreas, irregular narrowing of the MPD, serological findings, pathological findings, the presence of other IgG4-RDs, and the effectiveness of steroid therapy. Among other organ manifestations, IgG4-SC was the most frequently reported IgG4-RD (48.6%) in a Japanese nationwide survey on AIP [22].

### 3.2. Conventional EUS

**Figure 1:**
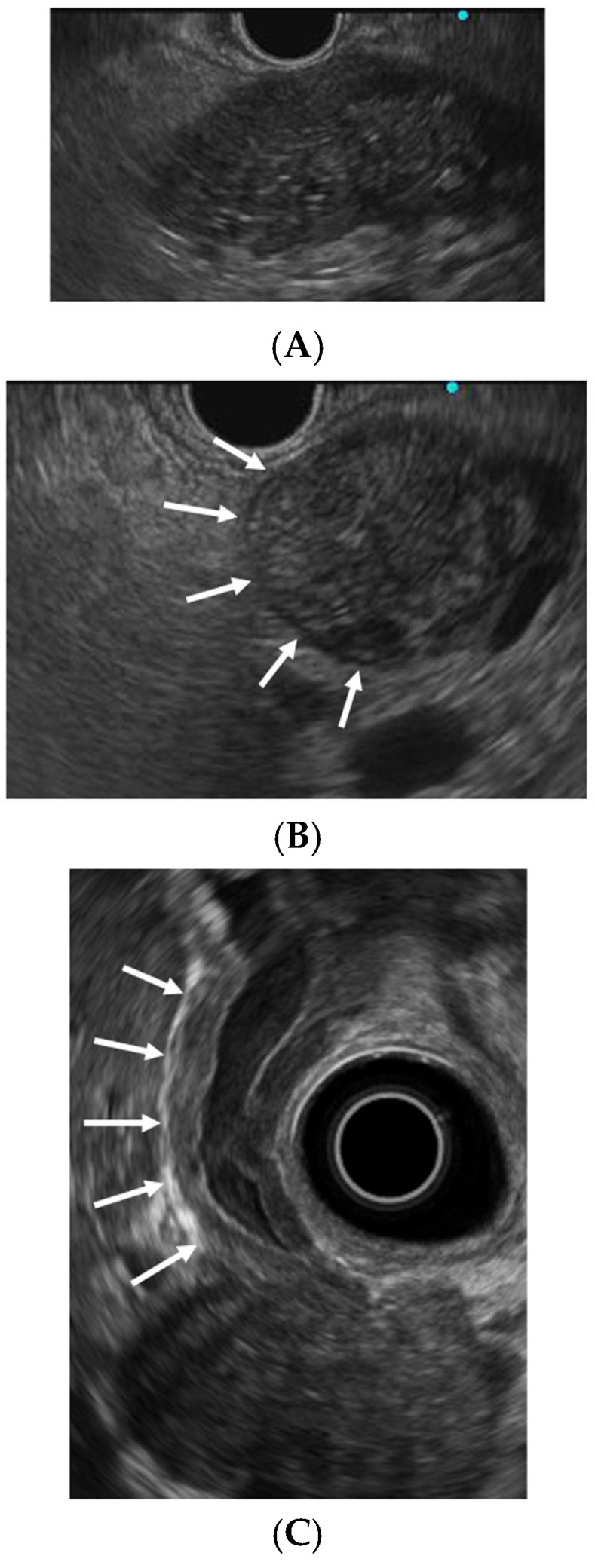
Endoscopic ultrasonographic findings of autoimmune pancreatitis and IgG4-related sclerosing cholangitis. (**A**) Diffuse pancreatic enlargement with a hypoechoic (“sausage-like”) appearance. (**B**) Peripancreatic hypoechoic margin (arrows). (**C**) Bile duct wall thickening (arrows).

EUS is used to assess pancreatic enlargement in the diagnosis of AIP. Characteristic conventional EUS findings include diffuse pancreatic enlargement with a hypoechoic (“sausage-like”) appearance, peripancreatic hypoechoic margins, and bile duct wall thickening (Figure 1) [8,13,23,24]. Focal pancreatic enlargement must be distinguished from PC. A notable differentiating feature is the duct-penetrating sign, defined as an uninterrupted MPD traversing the mass without obstruction (16). In a retrospective study of 285 patients with AIP, Zhan et al. [25] reported hypoechoic areas (74.7%), bile duct wall thickening (68.4%) or stenosis (57.9%), peripancreatic lymphadenopathy (31.2%), and peripancreatic hypoechoic margins (28.4%) as common findings on conventional EUS.

In the ICDC [15] and Japanese guidelines for AIP 2020 [7], EUS is recommended for the evaluation of parenchymal imaging, while MRCP and ERP are primarily used for the assessment of ductal imaging. In contrast, the European guideline emphasizes the use of EUS not only for parenchymal imaging but also for ductal imaging [14]. EUS plays a major role in the diagnostic algorithm in the European guideline.

### 3.3. Contrast-Enhanced Harmonic EUS

Contrast-enhanced harmonic EUS (CEH-EUS) can aid the differential diagnosis of AIP. Dong et al. [26] retrospectively reported focal or diffuse iso-enhancement patterns in the arterial phase in 86% of patients with AIP; a hypo-enhanced pattern was observed in 93.7% of those with PC. In the late phase, AIP was associated with hyper-enhanced (65%) or iso-enhanced (35%) patterns, whereas PC remained hypo-enhanced (93.7%). Cho et al. [27] found that focal AIP showed hyper- to iso-enhancement in the arterial phase (AIP: 89% vs. PC: 13%), homogeneous contrast distribution (AIP: 81% vs. PC: 17%), and absent irregular internal vessels (AIP: 85% vs. PC: 30%). Time-intensity curve analysis enables continuous and quantitative evaluation of enhancement patterns, and its usefulness in distinguishing AIP from PC has been reported [28,29,30].

### 3.4. EUS-Elastography

EUS elastography (EUS-EG) is a useful modality for evaluating pancreatic masses. Recently, EUS has incorporated shear wave elastography, which objectively measures tissue elasticity using absolute values. Dietrich et al. [31] reported that AIP exhibits a characteristically stiff elastographic pattern not only in the lesion but also in the surrounding pancreatic parenchyma. In a study by Ishikawa et al. [32], patients with AIP, including two without improvement in pancreatic enlargement after steroid therapy, showed a statistically significant decrease in strain ratio within 2 weeks of treatment. Ohno et al. [33] reported that the median shear wave velocity was significantly higher in an AIP group than in normal controls, and the mean velocity significantly decreased after steroid therapy. EUS shear wave elastography is a promising diagnostic tool for AIP due to its objectivity and quantifiability relative to conventional strain-based EUS-EG.

### 3.5. EUS-TA

**Figure 2:**
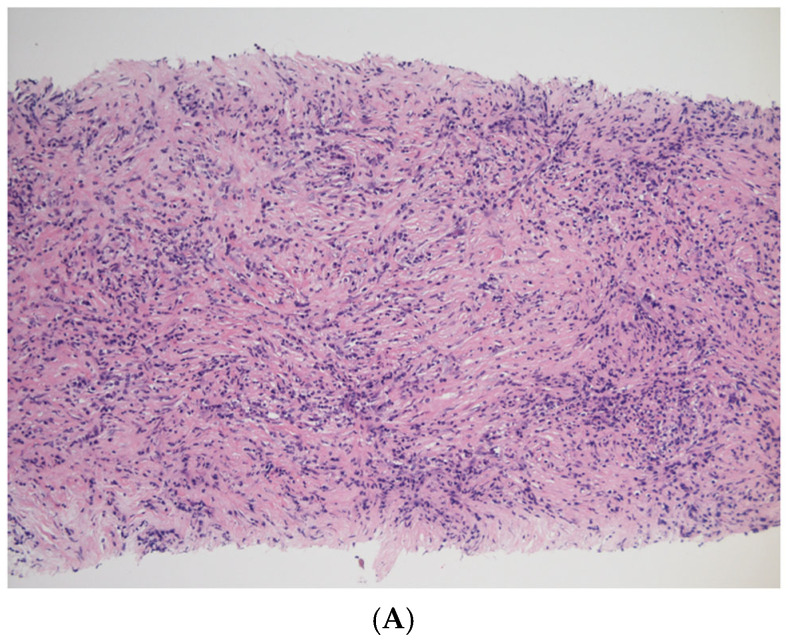

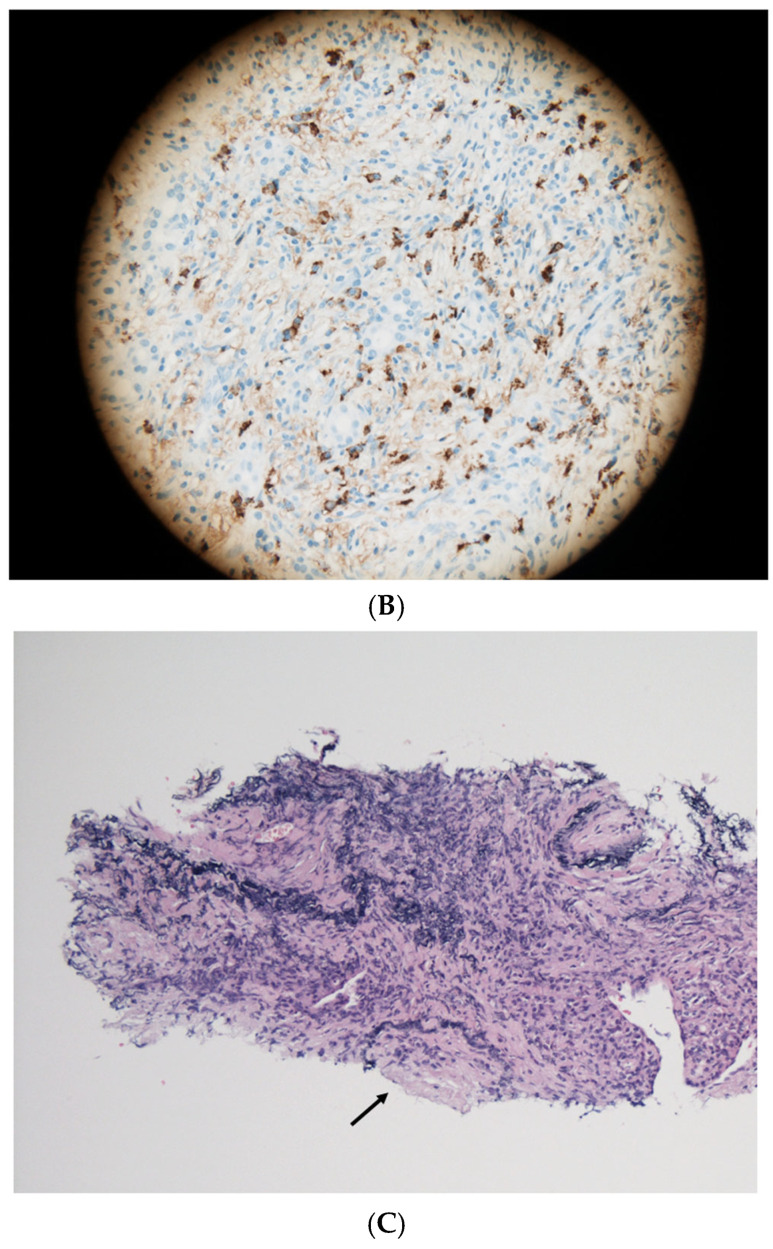
Pathological findings of autoimmune pancreatitis by endoscopic sonography-guided tissue acquisition. (**A**) Marked lymphoplasmacytic infiltration and storiform fibrosis (hematoxylin and eosin stain). (**B**) Abundant IgG4-positive plasma cells (immunostaining for IgG4). (**C**) Obliterative phlebitis (elastica masson stain). Arrow: obliterative phlebitis.

EUS-TA is used for the pathological diagnosis of AIP. Characteristic histologic features include marked lymphoplasmacytic infiltration, numerous IgG4-positive plasma cells, storiform fibrosis, obliterative phlebitis, and inflammatory cell infiltration around the duct epithelium (Figure 2). The ICDC [15] and JPS2018 [5] define key pathological features as lymphoplasmacytic infiltration with fibrosis, more than 10 IgG4-positive plasma cells per high-power field (HPF), storiform fibrosis, and obliterative phlebitis. A definitive pathological diagnosis requires at least three of these features. Exclusion of other pancreatic diseases, particularly PC, is a critical role of EUS-TA, and the absence of neoplastic cells on EUS-FNA is also included among the pathological criteria in JPS2018. The use of elastic stains and immunostaining for IgG4 and IgG is essential for establishing a histological diagnosis using biopsy specimens [34,35].

Although EUS-FNA was previously considered insufficient to acquire adequate tissue for the histological diagnosis of AIP, the advent and widespread adoption of EUS-FNB has substantially improved both specimen yield and quality [9]. Systematic reviews have shown the superiority of EUS-FNB over EUS-FNA according to the ICDC [15]. Chhoda et al. [36] reported that EUS-FNB yielded significantly higher specimen adequacy (96.8% vs. 79.8%, *p* = 0.016) and diagnostic sensitivity (60.2% vs. 42.0%, *p* < 0.0001). Facciorusso et al. [37] also demonstrated higher diagnostic accuracy for EUS-FNB than for EUS-FNA (63% vs. 45.7%, *p* < 0.001). In s study by Yoon et al. [38], the pooled diagnostic yield for level 1 or 2 histology criteria was significantly higher with EUS-FNB (87.2%) than with EUS-FNA (55.8%, *p* = 0.030). Consequently, EUS-TA using EUS-FNB is recommended for the pathological diagnosis of AIP. Various techniques for tissue acquisition during EUS-FNB of pancreatic masses have been reported, including slow-pull, dry suction, modified wet suction, and no suction methods [39,40]. Network meta-analysis revealed that modified wet suction seemed to provide high rates of integrity and adequate samples for solid pancreatic masses. However, to the best of our knowledge, techniques for tissue acquisition in autoimmune pancreatitis have not been systematically reported [41].

Several studies have evaluated needle size for EUS-FNB in AIP diagnosis according to the ICDC [15]. Kurita et al. [42] noted a significantly higher diagnostic yield for level 1 or 2 histology using a 22-gauge Franseen needle than using a 20-gauge forward-bevel needle (78% vs. 45%, *p* = 0.001). Tsutsumi et al. [43] reported successful diagnosis in nine of 14 patients (64%) using a 21-gauge Menghini-type needle. In a study by Ishikawa et al. [44], 16 of 20 patients (80%) were diagnosed using a 19-gauge Franseen needle. Iwata et al. [45] recently reported that the level 1 diagnostic rate was significantly higher with a 19-gauge FNB needle than with a 22-gauge FNB needle (90.3% vs. 61.1%, *p* = 0.010), and the larger needle yielded considerably larger tissue specimens. EUS-TA with a core biopsy, with a 19-gauge needle, is recommended in the European guideline on IgG4-related digestive diseases [12].

### 3.6. ERP

Diagnostic ERCP has been used for the diagnosis of AIP in Japan, but it is rarely utilized in Western countries. Magnetic resonance cholangiopancreatography (MRCP) is more commonly used due to its improved image quality and lower invasiveness. ERCP carries a risk of post-ERCP pancreatitis (PEP), which may result in serious complications. A recent systematic review of randomized controlled trials reported cumulative and severe PEP incidence rates of 10.2% and 0.5%, respectively [46]. A previous retrospective cohort study showed that the incidence of PEP was lower among patients with AIP than among controls (1.2% vs. 5.4%) [47]. Unnecessary ERCP should be avoided in the diagnosis of AIP and IgG4-SC, although the PEP risk may be lower in patients with AIP.

**Figure 3:**
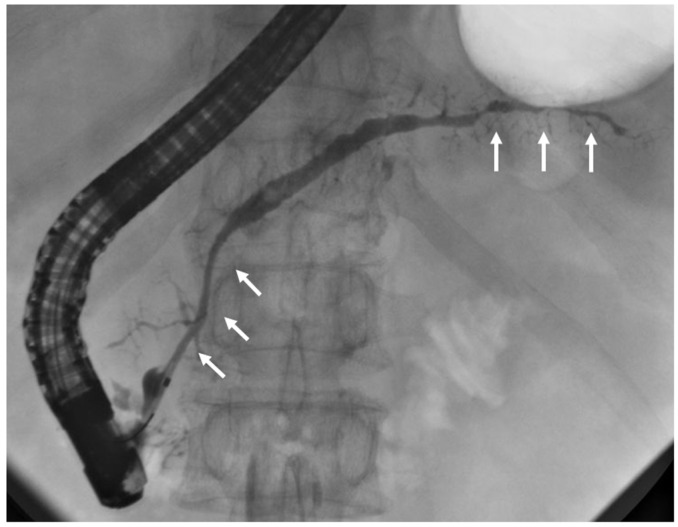
Endoscopic retrograde pancreatography showing the skipped narrowing of main pancreatic duct in the pancreatic head and tail. Side branches arise from main pancreatic duct narrowing. Arrows: skipped narrowing of main pancreatic duct.

A characteristic ERP finding in AIP is distinctive narrowing of the MPD [48,49,50,51,52]. Diffuse-type AIP demonstrates narrowing along more than one-third of the MPD. Long-segment or multiple skipped narrowings are specific pancreatographic features of AIP, and ERP is unnecessary when these are evident via MRCP. However, focal narrowing (less than one-third of the MPD) may be evident in focal-type AIP. ERP is useful in such cases, particularly when histologic evidence of AIP or malignancy exclusion via EUS-TA cannot be obtained. Key ERP features of AIP include narrowing along more than one-third of the MPD, multiple skipped narrowings, absence of upstream MPD dilation (<5 mm), and side branches arising from narrowed segments (Figure 3). In contrast, PC typically presents as a short, single MPD stricture with upstream dilation. Long or multiple skipped narrowing of MPD without marked upstream dilatation are defined as level 1 ductal imaging criteria in the ICDC [15]. In JPS2018, ERP findings were considered equivalent to a combination of MRCP and negative cytologic findings for malignancy via EUS-FNA.

### 3.7. Duodenal Papilla Biopsy

**Figure 4:**
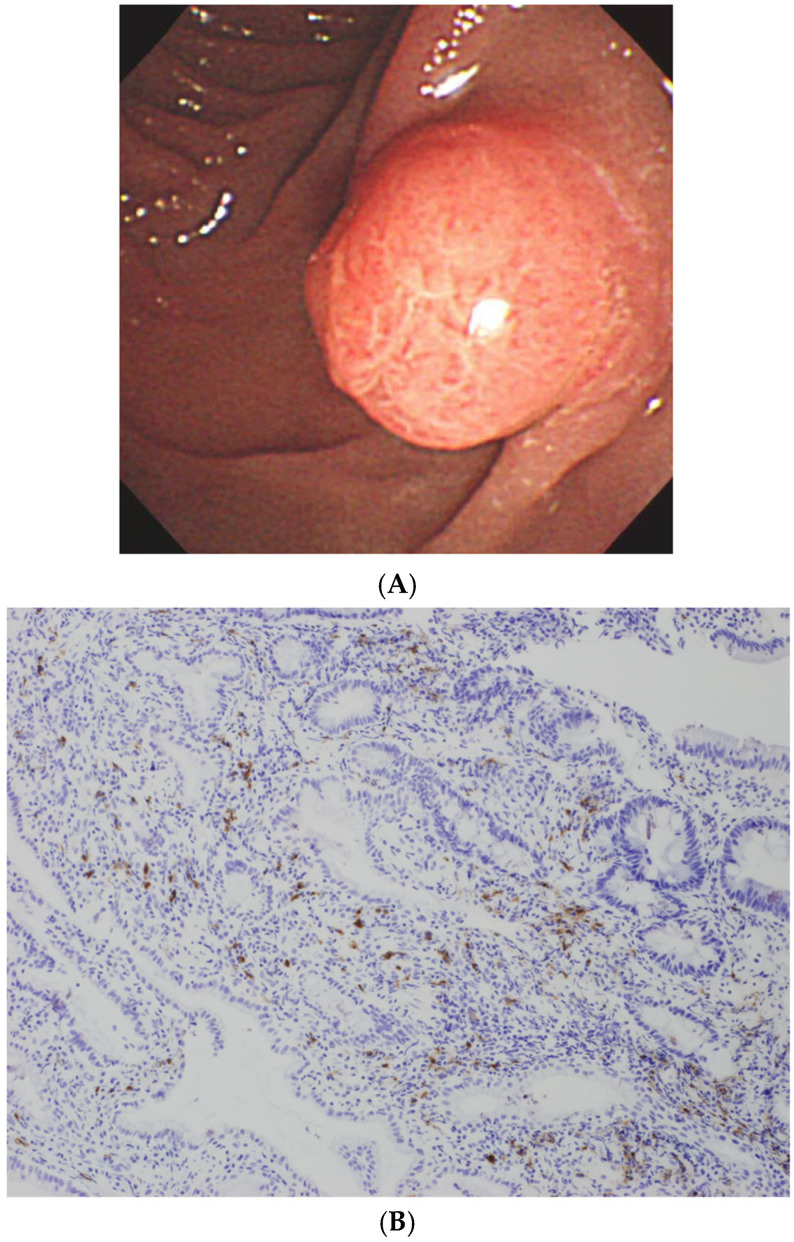
Endoscopic appearance and histological finding of duodenal papilla in AIP. (**A**) Swollen duodenal papilla. (**B**) Abundant IgG4-positive plasma cells in the biopsy specimen from duodenal papilla (immunostaining for IgG4).

Swollen duodenal papillae and abundant IgG4-positive plasma cells in biopsy specimens are observed particularly in cases with pancreatic head involvement (Figure 4). The international consensus diagnostic criteria include duodenal papilla biopsy as an optional diagnostic approach [15]. We previously reported that duodenal papilla biopsy was the most useful method for diagnosing AIP among comprehensive immunostaining evaluations conducted across eight different organs [53]. Furthermore, pancreatic head involvement, intrapancreatic IgG4-SC, and duodenal papilla swelling were significantly associated with positive IgG4 immunostaining in duodenal papilla samples. Pathological analysis of duodenal papilla biopsy specimens with IgG4 immunostaining serves as a valuable supplemental tool in diagnosing AIP [53,54,55,56,57] and helps to differentiate it from other mimickers.

## 4. IgG4-Related Sclerosing Cholangitis

### 4.1. Clinical Overview

IgG4-SC represents bile duct involvement of IgG4-RD and typically presents with biliary stricture and thickening of the bile duct wall on imaging [58,59]. Among extra-pancreatic IgG4-RDs, AIP is frequently associated with IgG4-SC. Primary sclerosing cholangitis (PSC) and cholangiocarcinoma (CCA) are important differential diagnoses. Two sets of diagnostic criteria have been proposed: the HISORt criteria developed in the United States [60], and the clinical diagnostic criteria for IgG4-SC 2012 (revised as the clinical diagnostic criteria for IgG4-SC 2020) established in Japan [4]. In Japan, the clinical diagnostic criteria for IgG4-SC 2020 (IgG4-SC2020) [6] and the clinical practice guidelines for IgG4-SC [3] are used in diagnosis of IgG4-SC. IgG4-SC2020 includes six criteria: narrowing of the intrahepatic and/or extrahepatic bile duct, thickening of the bile duct wall, serological findings, pathological findings, coexistence with other IgG4-RDs, and the effectiveness of steroid therapy. In a Japanese nationwide survey, AIP was the most commonly associated IgG4-RD among patients with IgG4-SC (83.7%) [61].

### 4.2. Cholangiographic Classification

**Figure 5:**
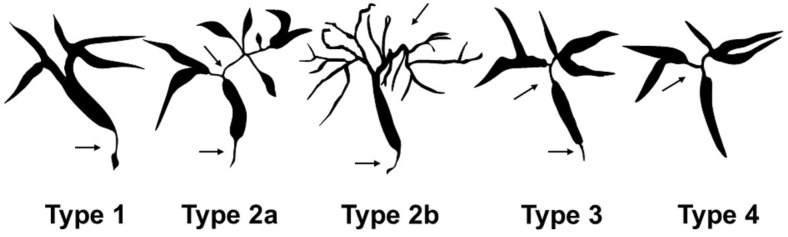
Cholangiographic classification of IgG4-related sclerosing cholangitis. Adapted with permission from Ref. [4]. 2012, John Wiley and Sons. Type 1. Stenosis is located only in the distal common bile duct. Type 2: Stenosis is diffusely distributed throughout the intrahepatic and extrahepatic bile ducts. Type 2a: Stricture of the intrahepatic bile ducts with prestenotic dilation. Type 2b: Stricture of the intrahepatic bile ducts without prestenotic dilation and reduced bile duct branches. Type 3: Stenosis is located in both the hilar hepatic and the distal common bile ducts. Type 4: Stenosis is located only in the hilar hepatic bile ducts. Arrows: Stenosis of the bile ducts.

IgG4-SC exhibits various cholangiogram and is classified into four types based on the location of biliary stricture [4] (Figure 5). Type 1 involves stricture limited to the distal bile duct and must be differentiated from PC, distal CCA, and chronic pancreatitis. Type 2 shows diffuse involvement of both intrahepatic and extrahepatic bile ducts; it resembles PSC. Type 2 is further subdivided into type 2a (with prestenotic dilation) and type 2b (without prestenotic dilation and with reduced bile duct branches). Type 3 presents with strictures in both the hilar hepatic and distal bile ducts. Type 4 involves strictures isolated to the hilar region. Types 3 and 4 require differentiation from hilar CCA. This classification, incorporated into IgG4-SC2020 [6], is essential for distinguishing IgG4-SC from other mimickers [62] and is featured in the diagnostic algorithm of the clinical practice guidelines [3]. In the Japanese nationwide survey [61], type 1 was the most common cholangiogram among all IgG4-SC cases (62.9%) and those associated with AIP (69.9%), whereas type 4 was most common among cases not associated with AIP (30.9%).

### 4.3. EUS

EUS is used to evaluate bile duct wall thickening in IgG4-SC. Detailed EUS findings of IgG4-SC have not been extensively reported. However, a nationwide survey in Japan revealed that bile duct wall thickening at non-stricture sites was observed in 73.8% of IgG4-SC cases [61]. Given its minimally invasive nature, EUS is well suited for assessment of bile duct wall thickening, a diagnostic item in IgG4-SC2020 (5). Because most IgG4-SC cases are associated with AIP, recognized in the diagnostic criteria as a key related IgG4-RD, EUS remains a valuable modality during IgG4-SC evaluation.

### 4.4. EUS-TA

EUS-TA can be considered when histological diagnosis is not achievable through transpapillary bile duct biopsy or cytology. Meta-analyses have demonstrated a high diagnostic yield of EUS-TA in malignant biliary strictures [63,64]. Matsumoto et al. [65] described a case of IgG4-SC diagnosed via EUS-TA after multiple inconclusive transpapillary biopsies. Although bile duct biopsy has low sensitivity in the diagnosis of IgG4-SC, EUS-TA may serve as a useful alternative for obtaining histological evidence.

### 4.5. ERC

MRCP is now commonly used instead of ERC to evaluate the cholangiogram in IgG4-SC because of its noninvasiveness and ability to assess both the cholangiogram and pancreatogram—important given the frequent association between AIP and IgG4-SC. However, ERC still offers superior image quality and is often used to perform additional ERC-related procedures or biliary drainage. Considering the risk of PEP, unnecessary ERC should be avoided, although PEP incidence is reportedly lower in patients with AIP.

When distinguishing IgG4-SC from malignancy, PC and CCA are key mimickers. A smooth biliary stricture without complete obstruction is a typical finding in IgG4-SC, whereas irregular strictures and complete obstruction suggest malignancy. Skipped strictures are also suggestive of IgG4-SC. In PC, distal bile duct strictures deviating leftward are frequently observed. PSC is a key condition in the differential diagnosis of type 2 IgG4-SC due to its similar cholangiographic patterns. ERC can differentiate IgG4-SC from PSC based on distinct cholangiographic features [66]. In IgG4-SC, strictures tend to be relatively long and are followed by simple dilation after a confluent narrowing. Intrapancreatic biliary strictures are common due to the association with AIP. In contrast, PSC is characterized by band-like (1–2 mm) strictures, beading, a pruned-tree appearance, and diverticulum-like outpouchings. Guidelines for diagnosing PSC, including those from Japanese, recommend MRCP as the initial imaging modality because its accuracy approaches that of ERC [67]. However, MRCP image quality can vary depending on equipment and patient-specific factors. ERC is not needed if MRCP yields adequate cholangiographic information.

### 4.6. IDUS

IDUS is a reliable procedure for bile duct evaluation during ERCP, providing high-resolution images that display the bile duct wall in three distinct layers. It should be performed before initial biliary drainage because drainage can cause mechanical inflammation. Bile duct wall thickening is included among the diagnostic criteria in IgG4-SC2020 [6]. Characteristic IDUS findings of IgG4-SC include circular, symmetrical wall thickening; smooth inner and outer margins; and a homogeneous internal echo at the stricture site [56,68,69,70,71]. In contrast, typical IDUS findings in PSC include circular, asymmetrical wall thickening; an irregular inner margin; unclear outer margin; diverticulum-like outpouchings; heterogeneous internal echo; and disappearance of the three layers [72]. IDUS finding at the stricture site is useful in the differentiation between IgG4-SC and PSC. These differences of IDUS findings between IgG4-SC and PSC are considered to reflect distinct underlying pathological alterations in the bile duct.

**Figure 6:**
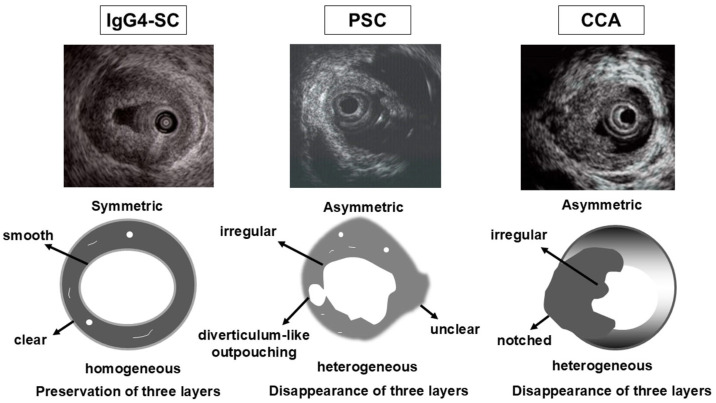
Intraductal ultrasonographic findings in the biliary stricture among IgG4-SC, PSC, and cholangiocarcinoma. CCA, cholangiocarcinoma; IgG4-SC, IgG4-related sclerosing cholangitis; PSC, primary sclerosing cholangitis.

Bile duct wall thickening at non-stricture sites is another characteristic IDUS finding for IgG4-SC. In IgG4-SC, wall thickening often extends continuously from the intrapancreatic to the hilar bile duct [56,68,72]. In contrast, such thickening is uncommon in CCA, making IDUS findings at non-stricture sites valuable for distinguishing IgG4-SC from CCA [68]. In a Japanese nationwide survey [61], bile duct wall thickening at non-stricture sites was detected by IDUS in 80.9% of IgG4-SC cases and was significantly more frequent than with EUS (80.9% vs. 73.8%, *p* = 0.045). These findings are summarized in Figure 6. However, as previously mentioned, caution is warranted due to the potential risks of ERCP-related adverse events.

### 4.7. Transpapillary Bile Duct Biopsy

Bile duct biopsy is performed to obtain histological evidence in indeterminate biliary strictures, particularly to exclude malignancy. Diagnostic features of IgG4-SC according to IgG4-SC2020 include lymphoplasmacytic infiltration, fibrosis, >10 IgG4-positive plasma cells per HPF, storiform fibrosis, and obliterative phlebitis [6]. However, the sensitivity of bile duct biopsy for detecting these features considerably varies (0–88%) [60,68,73,74]. In a nationwide Japanese survey (46), the rates of these histological features among bile duct biopsy samples were 32.9%, 16.9%, 0.6%, and 0.0%, respectively. The low sensitivity arises from the characteristic pathology: the bile duct epithelium is often normal, whereas diagnostic infiltration occurs in the subepithelial stroma, an area difficult to sample with small forceps.

CCA is a major mimicker of IgG4-SC, and bile duct biopsy plays a key role in ruling it out. A systematic review revealed a pooled sensitivity of 48.1% for detecting malignant biliary strictures [75]. Thus, bile duct biopsy is primarily useful for excluding malignancy rather than confirming IgG4-SC. Fluorescence in situ hybridization (FISH) has also been used to support diagnosis; in one study, applying FISH to transpapillary biopsy specimens increased sensitivity from 69.4% (hematoxylin and eosin only) to 77.6% [76].

### 4.8. Duodenal Papilla Biopsy

In a nationwide Japanese survey [61], swollen duodenal papillae and >10 IgG4-positive plasma cells per HPF were observed in 25.4% and 36.8% of IgG4-SC cases, respectively. This threshold was more frequently exceeded in duodenal papilla biopsies than in bile duct biopsies (36.8% vs. 16.9%). Given its simplicity and technical ease relative to bile duct biopsy, duodenal papilla biopsy serves as a valuable supplemental diagnostic tool, especially in cases with pancreatic head involvement.

## 5. IgG4-Related Cholecystitis

### 5.1. Clinical Overview

IgG4-related cholecystitis is a gallbladder (GB) manifestation of IgG4-RD. The term was formally introduced as part of IgG4-RD nomenclature in 2012 [77]. Whereas diffuse forms have been described in cohort studies [78], localized IgG4-related cholecystitis has mostly been reported in case series or individual reports [79,80,81,82,83,84]. Its clinicopathologic features have not yet been established through large cohort studies, and no formal diagnostic criteria currently exist. Differential diagnoses include GB cancer, adenomyomatosis (ADM), xanthogranulomatous cholecystitis (XGC), malignant lymphoma, and metastatic neoplasms [85]. GB wall thickening suggestive of diffuse IgG4-related cholecystitis was identified in 29% of AIP patients and 67% of IgG4-SC patients within a 258-case IgG4-RD cohort [78].

### 5.2. EUS

**Figure 7:**
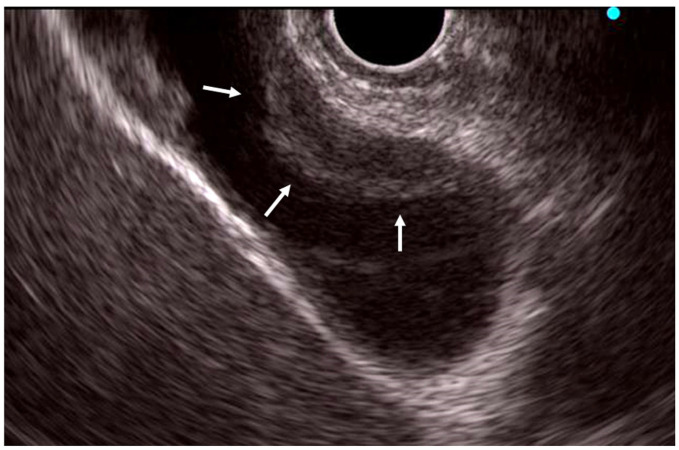
Endoscopic ultrasonographic findings showing a localized thickening of the gallbladder wall at the fundus, with the lesion located within the submucosal layer in localized IgG4-related cholecystitis (arrows).

EUS is useful for evaluating IgG4-related cholecystitis, which can present in diffuse or localized forms. Imaging features include continuity of the mucosal layer, a smooth luminal surface, and preservation of wall stratification. Localized IgG4-related cholecystitis may appear similar to subepithelial lesions (Figure 7), helping distinguish it from GB cancer. In some cases, cystic lesions within the GB wall, resembling ADM, have been reported in localized disease [85].

### 5.3. EUS-TA

EUS-TA is an effective diagnostic option for GB lesions, with reported sensitivity and specificity of 80–100% and 100%, respectively [86]. It is particularly useful for differentiating IgG4-related cholecystitis from mimickers such as GB cancer and XGC. Nagai et al. [87] and Wu et al. [88] each reported cases of IgG4-related cholecystitis mimicking GB cancer that were diagnosed via EUS-TA. This modality should be used cautiously and only for GB or bile duct lesions that do not require transgression of the bile duct lumen; such use will prevent bile leakage and lower cancer dissemination risk. Findings of endosonography-related procedures for IgG4-related pancreatobiliary diseases are summarized in Table 1.

### 5.4. Transpapillary GB Biopsy

Kawakami et al. [84] reported a case of localized IgG4-related cholecystitis diagnosed using transpapillary GB biopsy with a novel delivery system. Subsequent laparoscopic cholecystectomy confirmed the diagnosis. Transpapillary GB biopsy may be considered a diagnostic option in selected cases.

## 6. Conclusions

We reviewed the current roles of endoscopic modalities in diagnosing IgG4-related pancreatobiliary diseases. EUS and ERCP remain the two principal diagnostic modalities. EUS-related procedures—including conventional imaging, CEH-EUS, EUS-EG, and EUS-TA—are essential to detect both structural and histological features. EUS-TA is particularly important for obtaining pathological evidence. ERCP-related procedures, including IDUS, bile duct biopsy, and duodenal papilla biopsy, are useful for distinguishing IgG4-related diseases from PC, CCA, and PSC. Collectively, endoscopy plays a central role in the comprehensive diagnostic workup of IgG4-related pancreatobiliary diseases.

## Figures and Tables

**Table 1:** Findings of endosonography-related procedures.

Modality	AIP	IgG4-SC	IgG4-Related Cholecystitis
Conventional EUS	Hypoechoic pancreatic enlargement Peripancreatic hypoechoic margin Duct-penetrating sign	Wall thickening of bile duct	Diffuse/localized wall thickening of gallbladder Continuity of the mucosal layer Smooth luminal surface Preservation of wall stratification
Contrast-enhanced harmonic EUS	Hyper-iso enhancement pattern Homogeneous contrast distribution Absent irregular internal vessels	N/A	N/A
EUS-elastography	Stiff pattern in both mass and surrounding pancreatic parenchyma	N/A	N/A
EUS-TA	Marked lymphoplasmacytic infiltration and fibrosis >10 IgG4-positive plasma cells/HPF Storiform fibrosis Obliterative phlebitis		
IDUS	N/A	Circular-symmetrical wall thickening of bile duct Preservation of wall stratification Wall thickening at non-stricture site of bile duct	N/A

AIP, autoimmune pancreatitis; EUS, endoscopic ultrasonography; EUS-TA, endoscopic ultrasonography-guided tissue acquisition; HPF, high power field; IDUS, intraductal ultrasonography; IgG4-SC, IgG4-related sclerosing cholangitis; N/A, not available.

## Abbreviations

ADM; adenomyomatosis; AIP; autoimmune pancreatitis; CCA; cholangiocarcinoma; CEH-EUS; contrast-enhanced harmonic endoscopic ultrasound; ERC; endoscopic retrograde cholangioraphy; ERCP; endoscopic retrograde cholangiopancreatography; ERP; endoscopic retrograde pancreatography; EUS; endoscopic ultrasonography; EUS-EG; endoscopic ultrasonographic elastography; EUS-FNA; endoscopic ultrasonography-guided fine needle aspiration; EUS-FNB; endoscopic ultrasonography-guided fine needle biopsy; EUS-TA; endoscopic ultrasonography-guided tissue acquisition; GB, Gallbladder; HPF; high power field; ICDC; international consensus diagnostic criteria for autoimmune pancreatitis; IDUS, intraductal ultrasonography; IgG4-RD; IgG4-related disease; IgG4-SC; IgG4-related sclerosing cholangitis; IgG4-SC2020; clinical diagnostic criteria for IgG4-related sclerosing cholangitis 2020; JPS2018; Japanese clinical diagnostic criteria for autoimmune pancreatitis 2018; MPD; main pancreatic duct; PC; pancreatic cancer; PEP; post-endoscopic retrograde cholangiopancreatography pancreatitis; XGC; xanthogranulomatous cholecystitis.
